# Training the Trainer of an International Medical Graduate

**DOI:** 10.1192/bjo.2025.10309

**Published:** 2025-06-20

**Authors:** Akshith Shetty, Harleen Birgi, Ian Hall, Rahul Bhattacharya

**Affiliations:** 1 ELFT, London, United Kingdom.; 2 ELFT, l, United Kingdom

## Abstract

**Aims:** More than half of new recruits in the NHS are International Medical Graduates (IMGs). It is recognised that IMG need additional support, however to offer this their supervisors need to be aware of the landscape and resources to effectively support them. We developed a training for supervisors of IMGs using a mix of didactic, group work and simulation training.

**Methods:** The Faculty Development and IMG tutors surveyed and worked with several IMGs to identify topics for the training learning from their recent lived experiences. They also looked at guidance for IMG induction as published by General Medical Council (GMC) and British Medical Association (BMA). We identified that challenges of IMGs evolve through their journey and developed 3 simulation scenarios targeted at early, mid and later stages of the IMG pathway.

The one day face-to-face course included:

An introduction into current IMG landscape including the identified challenges. AS offered lived experience of IMG journey. This allowed for a discussion among the supervisors to reflect on their role.

This was followed by the 3 SIM scenarios:

An IMG doctor who had just moved to the UK and introduced to the supervisor the various practical hurdles that this entails around immigration and joining the NHS.

An IMG had been living in the UK for a few months and was starting a training job, with the focus on fleshing out the differences in healthcare systems.

Managing feedback about communication skills in an IMG who had significant clinical experience in their home country before moving to the UK a few years ago.

We gathered quantitative and qualitative feedback from the participants.

**Results:** 6 of the 7 candidates offered feedback which was unanimously positive. All found the content useful, most found the course extremely helpful to manage IMGs and an overall rating of excellent by 83% (5 out of 6). We received qualitative feedback as well, ‘It was amazing’ & ‘so grateful especially simulation rather than only theory’.

**Conclusion:** As IMGs enter the workforce through different pathways and at different times, organising focused IMG induction can be challenging. Moreover the needs of IMGs evolve over time. In psychiatry we have a structure of regular supervision which offers an opportunity of ongoing support to IMGs through the supervisors. However we believe supervisors are not always up to date with the IMG landscape or resources and would benefit from upskilling and updating in this field.

